# What Guides Visual Overt Attention under Natural Conditions? Past and Future Research

**DOI:** 10.1155/2013/868491

**Published:** 2013-12-12

**Authors:** Kai Kaspar

**Affiliations:** Social and Media Psychology, Department of Psychology, University of Cologne, Richard-Strauss-Straße 2, 50931 Cologne, Germany

## Abstract

In the last decade, overt attention under natural conditions became a prominent topic in neuroscientific and psychological research. In this context, one central question is “what guides the direction of gaze on complex visual scenes?” In the present review recent research on bottom-up influences on overt attention is presented first. Against this background, strengths and limitations of the bottom-up approach are discussed and future directions in this field are outlined. In addition to that, the current scope on top-down factors in visual attention is enlarged by discussing the impact of emotions and motivational tendencies on viewing behavior. Overall, this review highlights how behavioral and neurophysiological research on overt attention can benefit from a broader scope on influential factors in visual attention.

## 1. Introduction

Vision is the primary perceptual modality. Hence, visual information plays a key role in how we perceive the world and it constitutes the perceptual basis of our actions. The main task of the selection system is to choose the optimal location for the attention focus. This selection process is mediated by attention that bundles our limited cognitive resources and mediates an in-depth processing of environmental features at a given point in time [1, 2]. In this sense Posner [3] described the attentional focus in terms of a spotlight that scans the environment. In fact, the main task for the visual and oculomotor system is to direct one's gaze to the most important object in the visual field so that this object is projected to the fovea centralis providing sharp central vision. Given that the physiology of the human eye restricts the amount of relatively less noisy visual input at a certain point in time, one central question is “what guides the direction of gaze?” In this context it has to be pointed out that we commonly differentiate between several definitions and components of visual attention; on the one hand, we speak of sustained attention when we describe our readiness to detect rarely and abrupt occurring signals over prolonged periods of time [4]. By contrast, our ability to attend to one visual signal of current importance while ignoring others is defined as selective attention [5]. Moreover, we can also divide our attention to different locations in the visual field even when locations are spatially separated [6, 7]. Different models have been proposed which incorporate these different components of attentional processes [8, 9] as well as further components such as an alerting system that is involved in preparing and sustaining alertness to process signals of high priority [10]. In addition to these functional differences in attentional processes we traditionally differentiate between overt shifts of attention being paralleled by eye movements and covert shifts of attention being independent of eye movements. Although both are partially intertwined—for example, we cannot shift our gaze direction to locations being not attended [11]—there seems to be no controversy about the predominant role of gaze coupled, that is, overt shifts in visual attention. It is apparent that the later distinction does not substitute the classification previously described. For example, overt shifts of attention can be the result of either selective attention to a stimulus of current importance or a signature of sustained attention. Due to major achievements in neurophysiological research our knowledge about the biological basis of the different attentional processes continuously enlarges, for example, about the important role of the superior colliculus in the midbrain and the pulvinar in the thalamus in orienting behavior and their involvement in overt attention (for a current review see [10]).

In the following I will focus on overt attention and the factors influencing our gaze direction under natural conditions. Thereby the distinction between, for example, selective and sustained attention is secondary as both attentional modes are rather the result than the cause of those influential factors.

Within the large field of visual attention research a bulk of well-controlled experimental paradigms have been developed to systematically uncover the mechanisms of overt attention so far. In general, more or less artificial arrangements of simple geometric symbols, color cues, words, or images (such as cropped faces) are used to achieve a maximum of internal validity at the expense of external validity, that is, potential confound variables are eliminated to a high degree and this enables us to doubtlessly refer attentional effects to the treatment. However, in the last decade another experimental approach raised changing the tradeoff between internal and external, that is, ecological validity; in the last years techniques for recoding eye movements have made tremendous advances [12], facilitating the investigation of the spatiotemporal course of overt attention on static and dynamic complex scenes—from full-color computer-generated fractal images to urban and natural real-world scenes to movie clips. One of the main current objectives of attention research is to reveal those factors influencing eye movements on such complex scenes. Thereby, researchers are interested in both the quality of influential factors and the quantification of the individual impact of each factor. In the present review the current state of affairs as well as critical points for future research are outlined. On a very general level, it has been shown to be both useful and beneficial to refer to the fundamental distinction between stimulus-driven overt attention—also labeled as the *bottom-up* approach—and influences on attentional processes which are physiologically and functionally grounded beyond the primary visual system—also subsumed under the term *top-down* influences.

## 2. Strengths and Limitations of the Bottom-Up Approach

The visual cortex is hierarchically organized with higher specialization of neurons at later processing stages. Accordingly, models of visual perception have been formulated suggesting extraction from simple object features from the environment in early stages of visual processing until the integration of all these features at very late stages [13]. Such models suggested that the visual system works predominantly in a bottom-up way; that is, the visual input specifies the passive reaction of the visual system [14]. As a consequence researchers tried to explain variances in eye movements by elementary features of the visual scene, such as edges, direction of movement, disparity as well as contrasts in saturation, luminance, and color. The most prominent account is the idea of a saliency map [15, 16]; elementary features of the visual scene are extracted and separately represented by means of topographical cortical maps [17] at early stages in the visual processing timeframe. These feature maps preserve the neighborhood relations in the visual scene; that is, locations being spatially close together are mapped correspondingly [15]. Each of these maps indicates locations of the scene which are, compared to the surrounding, more striking. Thereby, different spatial scales are considered. At the end; that is, at higher visual areas, all feature maps are merged to one saliency map defining the overall conspicuity of scene locations. Several computational models were proposed which, in analogy to the processes in the neuronal substrate, create a saliency map for a given visual scene. Recent saliency map models sometimes predict actual eye movement by human subjects well [18]. However, although the quality of these predictions is about chance level [19], only small variance in fixation behavior can be explained by a saliency map in general [20]. In fact, even a very simple model that only considers the strong central bias of fixations on complex scenes would have some predictive value. Moreover, results were found contradicting the idea of a summation of separate feature maps but supporting the idea of bottom-up saliency maps in V1 in visual cortex [21]. Additionally, no evidence for a causal contribution of luminance, contrast—one important elementary scene feature—to a saliency map was found [20], questioning the validity of a universal saliency map. Hence several researchers focused on the impact of individual feature maps on fixation behavior in the last decade. Indeed, a bulk of studies revealed a significant influence of elementary scene features on fixation probability, for example, high spatial frequency [22], luminance [23, 24], edges [22, 25], and color [26]. The impact of such single features on gaze behavior does not evaporate over repeated scene observation [12], but scan patterns during a second observation of identical scenes revealed no correspondence with patterns predicted by saliency maps [27]. In general, researchers in this field seem to agree that the overall relationship of scene features and fixation likelihood is only modest [28–30]. Furthermore, the pure correlational nature of this relationship is pointed out [12, 19, 24, 28, 31, 32] because it is questionable whether elementary image features actually guide attention in a causal manner. It is conceivable that top-down operating factors primarily guide attention to specific locations in the visual field which coincide with visual saliency.

Despite these concerns, research on bottom-up impacts on attention revealed important insights into mechanisms of attention allocation under natural conditions. Indeed, we can imagine situations in which the visual attention is even completely stimulus driven, for example, when a stimulus abruptly enters the visual field [33] or when we make a compulsory look to a unique feature in the scene [34]. Evidence for stimulus-driven attention also comes from subjective reports; in a study where subjects were asked to verbally describe their impressions during repeated observation of identical static scenes, about 20% of subjects explicitly reported the impression that salient image regions or objects attracted attention automatically even after the scenes had become familiar to them [35].

## 3. What Can We Expect from Bottom-Up Oriented Research in the Future?

First of all, we can enlarge the scope on the correlation between elementary features of complex scenes and fixation likelihood; it is not only this correlation that is of interest but we can also set this measurement to other eye movement parameters. For example, Tatler et al. [36] found that short saccades (<8° visual angle) depend on elementary image features, but values (i.e., saliency) of such features at fixated locations are lower if long saccades precede. Moreover, this relationship between feature fixation correlations is revealed to be independent of scene familiarity and it is not a signature of target inaccuracy of the oculomotor system [12]: when we substitute the feature value of the actual fixation by the maximal feature value in a circular area of 1° around this fixation, the correlation between saccade length and feature value at the following fixation position (independent of the feature type, e.g., contrasts in luminance, saturation, or color) does not change. Such findings provide fruitful insights into the potential mechanism of saccade planning.

Second, feature fixation correlations as an indicator for bottom-up driven processes provide a basis for a better understanding of between-subject differences in eye movement behavior. Although neuroscientific research primarily focuses on mechanisms which are assumed to be universal, much variance often remains unexplained in studies. However, not all of this residual variance is a signature of noise and unsystematic fluctuations across subjects. Future research will benefit from explicitly considering interindividual differences, even when addressing the impact of stimulus features on the course of overt attention. For example, the above-mentioned correlation between image features at fixated locations and saccade length presumably interacts with systematic interindividual strategies regarding how to scan complex scenes. In a study where subjects repeatedly observed identical scenes, a clear distinction could be made between subjects who used long saccades to scan the scenes and those who used short saccades [12]. This distinction did not depend on the current presentation run of scenes. Importantly, subjects using short saccades in general showed an increasing coupling between saccades and elementary scene features (e.g., luminance contrast) over time. By contrast, subjects using long saccades showed higher feature-fixation correlations during the first observation of scenes, but these correlations decreased with increasing scene familiarity. Although the potential mechanism for this result is not completely understood so far, it indicates a new starting point for future research. A second example for the value of a combination of scene-feature-based research and research on individual differences was shown by Acik and colleagues [37]. They found that the impact of scene features on gaze behavior is age dependent. The attentional spotlight of young children (7–9 years) was heavily guided by elementary scene features, but in old adults (>72 years) image features correlated less with fixation behavior. Such findings contradict results of other studies showing that the weight of elementary scene features does not considerably vary across subjects [38]. However, intersubject variability can only be revealed when critical variables, such as subjects' age, are explicitly considered.

Third, Soltani and Koch [39] recently pointed out that, although research in the last years revealed much evidence for stimulus-driven allocation of attention, it has been unclear so far how this is accomplished at the neuronal level. In this context saliency-oriented computational approaches provide a suitable basis to investigate the mechanisms of stimulus-driven overt (and also covert) attention. In order to investigate the neuronal mechanisms of a saliency map they constructed a biophysically plausible network of spiking neurons simulating the formation of saliency signals in distinct cortical areas. Their network included three distinct layers of populations of neurons representing V1, V2, and V4, as well as one higher visual area instantiating a saliency map on the basis of individual feature maps represented at the lower visual areas. It is assumed that the frontal eye field [40] and the lateral intraparietal cortex [41] may be regarded as saliency maps. Soltani and Koch [39] found that lateral excitatory and inhibitory interactions in successive neuronal layers selective to individual features of a scene “provide a suitable mechanism for early saliency computations” (p. 12840). Moreover, with respect to Livingstone and Hubel [42], they conclude that neurons being specialized in the processing of single features, color and/or orientation, are more likely to contribute to bottom-up saliency in contrast to neurons being simultaneously selective to color and orientation. In turn, signals from lower layers can be improved by feedback from the layer that represents the saliency map. Consequently, such computational approaches provide new and important insights in biophysical mechanisms and constraints of saliency computations. Further work is necessary to better understand the neuronal mechanisms behind all the well-described psychological effects of bottom-up attention.

Fourth, although it seems to be common sense that a bulk of so-called top-down factors influence the course of overt attention on complex scenes dramatically, research on the impact of bottom-up attention has widely neglected this side of the coin. In the above-described study by Soltani and Koch [39], a first attempt was made to assess the impact of top-down signals within a neuronal network simulating the emergence of a saliency map. Based on the shown effect that signals from lower cortical layers were improved by feedback from the layer representing the saliency map, they assumed top-down attention to disrupt this feedback loop through its impact on the activity in the saliency map. Indeed, responses in V4 of monkeys showed that the saliency signal in V4 was eliminated when top-down attention was directed to a location far from the classical receptive field [43]. Hence, future research should also scrutinize the interplay between bottom-up and top-down influences on overt attention both on a behavioral level, that is, by using eye-tracking technology, and on the neuronal level by computational approaches, neurophysiological methods, and single cell recordings.

Finally, most of the insights into the impact of elementary scene features (and saliency maps) on attention allocation came from studies using static images. Static scenes provide time-independent arrangement and constancy of elementary scene statistics. Therefore, we do not have to make assumptions about dynamic changes in the spatial distribution of salient scene locations. However, with respect to a higher ecological validity of bottom-up driven overt attention motion is a central issue. Indeed, some models have been created proposing a computational principle for a motion saliency map (e.g., [44, 45]). For example, Mahapatra et al. [44] provided a model that identifies regions which are moving and salient, whereby the impact of motion on saliency is revealed to be stronger than the impact of other low-level features. Saliency by motion appears to be a key topic for future research, not only from the practical points of view as exemplarily shown by Ma et al. [45] who developed a model for automatic video summarization, but also regarding a better understanding of bottom-up driven attention in fully natural environments.

To conclude, research focusing on the bottom-up way of attention control revealed many important insights into those factors guiding our attentional spotlight. In this context, several directions for future research became apparent. However, some substantial concerns also arose, especially the limited variance in fixation behavior that can be explained by elementary scene features. Apparently, the impact of top-down influences is more than just an addition to bottom-up processes. This fact becomes visible, for example, when we compare the neuronal activity evoked by stimuli being similar in physical parameters but differing in their semantic content. In a high density electroencephalogram study by Kaspar et al. [46] images depicting either familiar or unfamiliar objects were foveally presented at a rate of 7.5 Hz, 12 Hz, or 15 Hz. These flickering rates elicited a continuous oscillatory brain response of the same frequency, the so-called steady state visual evoked potential (SSVEP). The results showed that at all three driving frequencies significant differences in the amplitudes of the corresponding SSVEPs occurred. These differences were localized to early occipital, lateral occipital, and temporal areas. Consequently, the semantic content of physically similar images can lead to very different responses in the human brain, indicating that top-down influences on visual processing are strong.

All in all, the bottom-up approach has provided important insight into the nature of overt attention on complex scenes, on both empirical and theoretical levels. However, there are some open questions which should be addressed in future studies.

- To what degree can top-down processes modulate (i.e., reweight) individual features of a saliency map? Are there specific features which are more susceptible to (certain) top-down influences (e.g., emotional processes, current motivation, and task)?
- How variable is the weighting of individual image features across different image categories? Although there are systematic differences in scene statistics across categories [47], some empirical evidence suggest that fast scene recognition may depend upon more semantic global information [48]. This indicates that top-down processes are directly involved in fast scene recognition and hence (partially) determine at very early stages the weighting of scene features in a saliency map. As Nystrom and Holmqvist [29] showed, contrast manipulations' impact on fixation selection heavily depends on the semantic content of the scene. They concluded that the impact of top-down factors, such as semantics, can easily override the impact of bottom-up factors in fixation selection. Similar results come from the field of face detection. In general, faces with negative facial expression are detected faster than positive faces, and emotional faces are detected faster than neutral faces, but this effect disappears when faces are rotated by 180° [49, 50] indicating that the emotional category of the stimulus rather than physical features is responsible for these effects.
- What is the relative impact of saliency by motion compared to saliency by static image properties (e.g., edges or contrast of color)? Furthermore, the traditional way by which the values of certain image features on certain scene locations is computed cannot be easily transferred to nonstatic scenes. Feature values at specific locations are commonly quantified by comparing these locations with the surrounding regarding the feature of interest. In this sense, these values are not absolute but relative. Given the fact that we actively move through the environment, the appearance of the visual scene continuously changes in the eye of the observer and consequently feature values must be updated dynamically. Against this background, the ecological validity of the results revealed by studies using real-world but static scenes is questionable to some (not quantifiable) degree.
- Besides these concerns the ultimate question is whether elementary scene features actually guide overt attention in a causal way. The pure correlational nature of feature-fixation relationships has been pointed out several times [12, 19, 24, 28, 31, 32]. Research would substantially benefit from experimental paradigms enabling us to disentangle the potential confound between pure visual saliency in terms of elementary scene features and saliency derived from top-down factors. As already mentioned, it is conceivable that top-down operating factors primarily guide attention to specific locations in the visual field which coincide with visual saliency. In other words, what remains when extracting all saliency defined by top-down processes?

## 4. Some Remarks on Top-Down Factors in the Context of Complex Scene Observation

The idea that top-down factors can influence eye movements on complex scenes is not novel but the primary focus is often limited to the impact of the current task (e.g., [51–53]). Accordingly, Soltani and Koch [39] speak in terms of a top-down effect of the task when referring to the effect of saccade preparation on the activity in the saliency map. However, Betz et al. [54] showed that substantial task-dependent changes in viewing behavior were paralleled by only minor changes in the correlation between fixation likelihood and elementary image features. Hence, top-down factors are not necessarily intertwined with bottom-up processes which are insufficient to explain the whole variance in attention allocation. In order to be able to explain much more variance it is necessary to widen the currently narrow scope on top-down factors.

Of special interest in this context are emotions. The concept of emotion is ambiguously defined [2], but one widely used theoretical framework postulates two independent dimensions specifying the range of emotions: arousal and valence [55]. This framework is partially supported by neurophysiological findings showing that anterior temporal areas and extrastriate visual cortex are activated independently by valence, arousal, and attention [56]. However, there is also much literature suggesting that both dimensions are rather strongly intertwined, either derived from theoretical considerations [57–59] or based on physiological analyses at different levels (autonomic, cortical, and endocrine) indicating that arousal includes multidimensional processing [60]. As a consequence, currently several functional pathways for an interaction between attentional and emotional processes are in the focus of research, but overall research in this field is just at the beginning and currently suffers from the diversity of concepts of both attention and emotion. For a detailed review about emotion-attention interaction on the neuronal and behavioral level see [2]. Although the neuronal mechanisms are poorly understood so far, evidence for a significant impact of emotions on attention is convincing at the behavioral level. With respect to human viewing behavior on complex scenes we found that several eye movement parameters, such as the duration of fixations, the saccade length, and the extent of visuospatial exploration, are very sensitive to the valence of the emotional context [61]; that is, while the visual configuration of elementary image features was constant, viewing behavior changed dramatically depending on the current emotional context. Thereby the intensity of the emotional context and the continuity of a specific valence over time moderated the effect. Apparently, the characteristics of the emotional context define the strength of the induced valence-congruent internal mood in the observer. Importantly, it is not only this internally located impact of emotion (i.e., the emotional state of the observer), but also the valence of the scene (i.e., the externally located impact of emotion) that modifies human gaze behavior in a top-down manner [2]. Until now, models of saliency maps and research focusing on elementary image features have not considered emotional components. It is a challenging task for future research to incorporate bottom-up factors and emotion as a strong top-down factor.

A second high-level factor being worth considering is the interaction between subjective scene interpretation and motivational disposition in terms of time-independent personality traits. In personality psychology there is a strong consensus that current motivation is always the result of the interplay between motivational dispositions (e.g., a tendency to behave or react in an extroverted or neuroticistic fashion) and the current situation [62]. Consequently, if we know what kind of interpretation people can extract from a visual scene and if we also know something about personal behavioral tendencies, we will be able to better understand why the course of overt attention differs so much between subjects. This will allow us to explain much more variance in observable gaze behavior that is commonly labeled as residual noise. Indeed, two recent eye-tracking studies showed that motivational dispositions substantially modified the way in which subjects scanned complex images [35, 63]. The consideration of individual scene interpretation—for example, boring versus interesting and stimulating—is necessary in this context in order to scrutinize the boundary conditions for strong motivational impacts on attention. In addition to the behavioral level, the consideration of motivational tendencies can also complete our understanding of neuronal mechanisms contributing to attention control. For example, extraversion, but not neuroticism, was found to be correlated with subcortical (caudate nucleus and putamen) rather than cortical brain regions [64]. The amount of subjects' extraverted behavioral tendencies did not correlate with the regional cerebral blood flow in the prefrontal, orbitofrontal, temporopolar, cingulate, and primary visual cortex. However, Rauthmann et al. [63] showed that extraversion was significantly related to eye movement parameters. From that point of view, attention should also be paid when speaking of top-down versus bottom-up impacts on attention. It is questionable whether we should speak of top-down influences on attentional processes when the neuronal basis of the influential factor (i.e., extraversion) is located in subcortical regions. A clear linguistic distinction should be made between bottom-up processes in terms of stimulus-driven attention, on the one hand, and bottom-up influences regarding the hierarchically organized brain, on the other hand. Moreover, it has been suggested that the traditional view of a hierarchically organized brain is partially inaccurate because the brain is comprised of parallel circuits [65], and hence description of neuronal processes in terms of bottom-up and top-down is probably misleading in general.

All in all, the consideration of personality traits and their interaction with the situational context requires an idea of how a main effect of personality traits and the interaction with situational factors could affect eye movement behavior in complex scenes. More specifically, the following three aspects are important.

1. First of all, the selection of the stimulus material depends on the aim that we pursue; if we want to reliably and validly distinguish subjects according to their differences in eye movements we do not need real-world scenes. Artificial arrangements of stimuli can be optimized to discriminate interindividual differences in traits on the basis of specific eye movement parameters as previously shown [63]. This strategy will be the first choice if we are interested in diagnostics and, indeed, it should be the first step in order to detect those image properties and statistics providing the highest diagnostic value. In the second step, we can infer to categories of real-world scenes which systematically differ in such scene statistics [47]. This procedure would lead to more precise predictions about the potential impact of certain traits on the observation of certain scene types. Moreover, we can assess the extent to which specific eye movement parameters (e.g., saccade frequency, saccade length, or dwell time) are susceptible for trait influences.
2. Second, we need an idea of how personality traits influence eye movement behavior over time. It is possible that (some) traits influence the absolute level of a parameter—for example, the mean saccade frequency in a given time interval—independently of the situational context. It is also possible that only specific contexts, that is, visual scenes, trigger an influence of personality traits. If so, the next question is in which direction the effect aims (e.g., increasing versus decreasing saccade frequency). At the moment we only can speculate the diversity of mechanisms as we do not exactly know which eye movement parameters are controlled simultaneously by a unique system and which parameters are processed independently. Furthermore, we have only little knowledge about the interaction between, on the one hand, those neuronal systems building the scaffold for specific personality traits and, on the other hand, potential neuronal interactions with those system(s) processing saccade parameters.
3. Third, it is important to note that the relationship between personality traits and the control of overt attention is not necessarily unidirectional. It is conceivable that habits in observing specific scenes are learned very early in life and form, at least partially, motivational dispositions and personal inclinations. However, it is hard to imagine how this could be experimentally tested.
4. Finally, although personality traits are stable over a long period of several years or even decades [66], some evidence suggests that there are changes in personality traits over the life span [67]. Hence, age should be considered as a covariate because it may affect the manifestation of specific traits.

In this context of time-dependent changes a time-scale oriented categorization of factors influencing attention control will be useful in general. Such a categorization will help to understand when, how long, and how strong the effect of a factor on eye movements can be. For example, a look to the above-described phenomena of stimulus-driven attention shows that a stimulus abruptly entering the visual field [33] automatically attracts attention. This effect is strong and hard to resist. By contrast, when we observe a static scene, the impact of elementary features does not substantially change over time and is only modest overall [12]. These two effects differ remarkably on the time scale. Within emotional influences we can also differentiate between effects on the time-scale; the emotional content of complex scenes can be fast recognized [61], and hence it can unfold its influence on overt attention immediately. Besides this first stimulus-driven attentional response, physiological responses within the observer occur with some temporal delay but maintain a high arousal level for a longer interval. This may lead to a long-lasting impact of emotions on viewing behavior. In other words, the externally located impact of emotion becomes internally located (c.f. [2, 61]). A time-scale orientation is also suitable for the impact of motivational dispositions which are stable over a long period of several years or even decades [66]. In this sense it is the most long-lasting factor producing systematic interindividual differences on eye movement behavior. However, the strength of motivational dispositions' impact on attention fluctuates depending on current situational factors (c.f. [35]). Hence, the variable of primary interest is the longevity of the situation as the situation is the trigger (behavioral and neuronal) for motivational effects on visual attention.

## 5. Conclusion

The visual sense plays an outstanding role in human perception and attention; that is, the bundling of limited cognitive resources allows in-depth processing of the currently most important location in the environment. In this context, one key question is “what guides the direction of gaze?” intensive research on the role of elementary scene features on fixation behavior showed that these features have a constant and time-independent influence. However, the correlational nature and the overall modest high of feature-fixation correlations call the utility of the bottom-up way into question. Nonetheless and as outlined in the preceding sections, this approach has great potential to stimulate future research with respect to other eye movement parameters, systematic intersubject variability, modeling of neuronal saliency computations, incorporation of high-level factors, and saliency by motion. Despite this great potential, research that limits its scope to exclusively stimulus-driven overt attention is insufficient to explain much of the variance in eye movement behavior across and within subjects. The consideration of task effects, of emotional components, internally and externally located, and of interplay between personality traits and situational factors is suitable to increase our understanding of visual attention at both the behavioral and the neurophysiological level. In this context, it appears useful to consider the individual time-scale of the different processes in order to get a better estimation of the point in time when an effect can be expected at the attentional level and how long-lasting this effect will be.

## References

[1] Chun MM, Golomb JD, Turk-Browne NB (2011). A Taxonomy of external and internal attention. *Annual Review of Psychology*.

[2] Kaspar K, Konig P (2012). Emotions and personality traits as high-level factors in visual attention. *Frontiers in Human Neuroscience*.

[3] Posner MI (1980). Orienting of attention. *The Quarterly Journal of Experimental Psychology*.

[4] Sarter M, Givens B, Bruno JP (2001). The cognitive neuroscience of sustained attention: where top-down meets bottom-up. *Brain Research Reviews*.

[5] Portas CM, Rees G, Howseman AM, Josephs O, Turner R, Frith CD (1998). A specific role for the thalamus in mediating the interaction of attention and arousal in humans. *Journal of Neuroscience*.

[6] Hahn S, Kramer AF (1998). Further evidence for the division of attention among non-contiguous locations. *Visual Cognition*.

[7] Müller MM, Malinowski P, Gruber T, Hillyard SA (2003). Sustained division of the attentional spotlight. *Nature*.

[8] Corbetta M, Shulman GL, Posner MI (2012). Two attentional networks: identification and function within a larger cognitive architecture. *Cognitive Neuroscience of Attention*.

[9] Coull JT (1998). Neural correlates of attention and arousal: insights from electrophysiology, functional neuroimaging and psychopharmacology. *Progress in Neurobiology*.

[10] Posner MI, Petersen SE (1990). The attention system of the human brain. *Annual Review of Neuroscience*.

[11] Hoffman JE, Subramaniam B (1995). The role of visual attention in saccadic eye movements. *Perception & Psychophysics*.

[12] Kaspar K, König P (2011). Viewing behavior and the impact of low-level image properties across repeated presentations of complex scenes. *Journal of Vision*.

[13] Biederman I (1987). Recognition-by-components: a theory of human image understanding. *Psychological Review*.

[14] Engel AK, Fries P, Singer W (2001). Dynamic predictions: oscillations and synchrony in top-down processing. *Nature Reviews Neuroscience*.

[15] Koch C, Ullman S (1985). Shifts in selective visual attention: towards the underlying neural circuitry. *Human Neurobiology*.

[16] Itti L, Koch C (2001). Computational modelling of visual attention. *Nature Reviews Neuroscience*.

[17] Zeki SM (1978). Functional specialisation in the visual cortex of the rhesus monkey. *Nature*.

[18] Harel J, Koch C, Perona P, Scholkopf B, Platt J, Hoffman T (2007). Graph-based visual saliency. *Advances in Neural Information Processing Systems 19*.

[19] Foulsham T, Underwood G (2008). What can saliency models predict about eye movements? Spatial and sequential aspects of fixations during encoding and recognition. *Journal of Vision*.

[20] Parkhurst D, Law K, Niebur E (2002). Modeling the role of salience in the allocation of overt visual attention. *Vision Research*.

[21] Koene AR, Zhaoping L (2007). Feature-specific interactions in salience from combined feature contrasts: evidence for a bottom-up saliency map in V1. *Journal of Vision*.

[22] Mannan SK, Ruddock KH, Wooding DS (1997). Fixation patterns made during brief examination of two-dimensional images. *Perception*.

[23] Krieger G, Rentschler I, Hauske G, Schill K, Zetzsche C (2000). Object and scene analysis by saccadic eye-movements: an investigation with higher-order statistics. *Spatial Vision*.

[24] Einhäuser W, König P (2003). Does luminance-contrast contribute to a saliency map for overt visual attention?. *European Journal of Neuroscience*.

[25] Baddeley RJ, Tatler BW (2006). High frequency edges (but not contrast) predict where we fixate: a Bayesian system identification analysis. *Vision Research*.

[26] Frey H-P, König P, Einhäuser W (2007). The role of first- and second-order stimulus features for human overt attention. *Perception & Psychophysics*.

[27] Underwood G, Foulsham T, Humphrey K (2009). Saliency and scan patterns in the inspection of real-world scenes: eye movements during encoding and recognition. *Visual Cognition*.

[28] Einhäuser W, Spain M, Perona P (2008). Objects predict fixations better than early saliency. *Journal of Vision*.

[29] Nystrom M, Holmqvist K (2008). Semantic override of low-level features in image viewing—both initially and overall. *Journal of Eye Movement Research*.

[30] Tatler BW, Baddeley RJ, Gilchrist ID (2005). Visual correlates of fixation selection: effects of scale and time. *Vision Research*.

[31] Henderson JM, Brockmole JR, Castelhano MS, Mack M, van Gompel R, Fischer M, Murray W, Hill R (2007). Visual saliency does not account for eye movements during search in real-world scenes. *Eye Movements: A Window on Mind and Brain*.

[32] Tatler BW (2007). The central fixation bias in scene viewing: selecting an optimal viewing position independently of motor biases and image feature distributions. *Journal of Vision*.

[33] Yantis S, Jonides J (1984). Abrupt visual onsets and selective attention: evidence from visual search. *Journal of Experimental Psychology: Human Perception and Performance*.

[34] Treisman AM, Gelade G (1980). A feature-integration theory of attention. *Cognitive Psychology*.

[35] Kaspar K, König P (2011). Overt attention and context factors: the impact of repeated presentations, image type, and individual motivation. *PLoS ONE*.

[36] Tatler BW, Baddeley RJ, Vincent BT (2006). The long and the short of it: spatial statistics at fixation vary with saccade amplitude and task. *Vision Research*.

[37] Acik A, Sarwary A, Schultze-Kraft R, Onat S, Konig P (2010). Developmental changes in natural viewing behavior: bottom-up and top-down differences between children, young adults and older adults. *Frontiers in Psychology*.

[38] Zhao Q, Koch C (2011). Learning a saliency map using fixated locations in natural scenes. *Journal of Vision*.

[39] Soltani A, Koch C (2010). Visual saliency computations: mechanisms, constraints, and the effect of feedback. *Journal of Neuroscience*.

[40] Schall JD, Thompson KG (1999). Neural selection and control of visually guided eye movements. *Annual Review of Neuroscience*.

[41] Gottlieb JP, Kusunoki M, Goldberg ME (1998). The representation of visual salience in monkey parietal cortex. *Nature*.

[42] Livingstone MS, Hubel DH (1987). Psychophysical evidence for separate channels for the perception of form, color, movement, and depth. *Journal of Neuroscience*.

[43] Burrows BE, Moore T (2009). Influence and limitations of popout in the selection of salient visual stimuli by area V4 neurons. *Journal of Neuroscience*.

[44] Mahapatra D, Winkler S, Yen S-C Motion saliency outweighs other low-level features while watching videos.

[45] Ma Y-F, Lu L, Zhang H-J, Li M A user attention model for video summarization.

[46] Kaspar K, Hassler U, Martens U, Trujillo-Barreto N, Gruber T (2010). Steady-state visually evoked potential correlates of object recognition. *Brain Research*.

[47] Torralba A, Oliva A (2003). Statistics of natural image categories. *Network: Computation in Neural Systems*.

[48] Velisavljevic L, Elder JH (2002). What do we see in a glance?. *Journal of Vision*.

[49] Eastwood JD, Smilek D, Merikle PM (2001). Differential attentional guidance by unattended faces expressing positive and negative emotion. *Perception & Psychophysics*.

[50] Lamy D, Amunts L, Bar-Haim Y (2008). Emotional priming of pop-out in visual search. *Emotion*.

[51] Triesch J, Ballard DH, Hayhoe MM, Sullivan BT (2003). What you see is what you need. *Journal of Vision*.

[52] Rothkopf CA, Ballard DH, Hayhoe MM (2007). Task and context determine where you look. *Journal of Vision*.

[53] Hamborg K-C, Bruns M, Ollermann F, Kaspar K (2012). The effect of banner animation on fixation behavior and recall performance in search tasks. *Computers in Human Behavior*.

[54] Betz T, Kietzmann TC, Wilming N, König P (2010). Investigating task-dependent top-down effects on overt visual attention. *Journal of Vision*.

[55] Kensinger EA (2004). Remembering emotional experiences: the contribution of valence and arousal. *Reviews in the Neurosciences*.

[56] Lane RD, Chua PM-L, Dolan RJ (1999). Common effects of emotional valence, arousal and attention on neural activation during visual processing of pictures. *Neuropsychologia*.

[57] Lang PJ (1994). The varieties of emotional experience: a meditation on James-Lange theory. *Psychological Review*.

[58] Eysenck HJ, Eysenck HJ (1981). General features of the model. *A Model for Personality*.

[59] Gray JA, Eysenck HJ (1981). A critique of Eysenck's theory of personality. *A Model for Personality*.

[60] Derryberry D, Rothbart MK (1988). Arousal, affect, and attention as components of temperament. *Journal of Personality and Social Psychology*.

[61] Kaspar K, Hloucal TM, Kriz J (2013). Emotions' impact on viewing behavior under natural conditions. *PloS ONE*.

[62] Heckhausen J, Heckhausen H (2006). *Motivation und Handeln*.

[63] Rauthmann JF, Seubert CT, Sachse P, Furtner MR (2012). Eyes as windows to the soul: gazing behavior is related to personality. *Journal of Research in Personality*.

[64] Fischer H, Wik G, Fredrikson M (1997). Extraversion, neuroticism and brain function: a pet study of personality. *Personality and Individual Differences*.

[65] Steinberg BA, Augustine JR (1997). Behavioral, anatomical, and physiological aspects of recovery of motor function following stroke. *Brain Research Reviews*.

[66] Cobb-Clark DA, Schurer S (2012). The stability of big-five personality traits. *Economics Letters*.

[67] Donnellan MB, Lucas RE (2008). Age differences in the big five across the life span: evidence from two national samples. *Psychology and Aging*.

